# Effects of *Nosema apis*, *N. ceranae*, and coinfections on honey bee (*Apis mellifera*) learning and memory

**DOI:** 10.1038/srep22626

**Published:** 2016-03-10

**Authors:** Lise R. Charbonneau, Neil Kirk Hillier, Richard E. L. Rogers, Geoffrey R. Williams, Dave Shutler

**Affiliations:** 1 Department of Biology, Acadia University, Wolfville, NS, Canada, B4P 2R6; 2 Bayer CropScience Ecotoxicology, 2 T. W. Alexander Drive, Research Triangle Park, NC, USA, 27709; 3 Department of Biology, Dalhousie University, Halifax, NS, Canada, B3H 4R2

## Abstract

Western honey bees (*Apis mellifera*) face an increasing number of challenges that in recent years have led to significant economic effects on apiculture, with attendant consequences for agriculture. Nosemosis is a fungal infection of honey bees caused by either *Nosema apis* or *N. ceranae*. The putative greater virulence of *N. ceranae* has spurred interest in understanding how it differs from *N. apis*. Little is known of effects of *N. apis* or *N. ceranae* on honey bee learning and memory. Following a Pavlovian model that relies on the proboscis extension reflex, we compared acquisition learning and long-term memory recall of uninfected (control) honey bees versus those inoculated with *N. apis*, *N. ceranae*, or both. We also tested whether spore intensity was associated with variation in learning and memory. Neither learning nor memory differed among treatments. There was no evidence of a relationship between spore intensity and learning, and only limited evidence of a negative effect on memory; this occurred only in the co-inoculation treatment. Our results suggest that if *Nosema* spp. are contributing to unusually high colony losses in recent years, the mechanism by which they may affect honey bees is probably not related to effects on learning or memory, at least as assessed by the proboscis extension reflex.

Western honey bees (*Apis mellifera*) are ecologically and economically important pollinators world-wide, with pollination services contributing billions of dollars annually^1^,^2^. For reasons that appear multi-causal^3^,^4^,^5^,^6^, honey bee colonies have in recent years been suffering significant mortality in regions around the world, likely at an unprecedented rate^7^. Causes of mortality include pesticides, shortages of forage, improper management by beekeepers, and parasites^8^,^9^,^10^,^11^,^12^,^13^,^14^,^15^. Among the latter are two species of microsporidian fungi, *Nosema apis* and *N. ceranae*. Although many signs of pathology have been identified for infections with *N. apis* and *N. ceranae*, effects of these parasites on honey bee acquisition learning (hereafter, learning) and long-term memory recall (hereafter, memory) are not well studied. Wright (ref. 16) found that fungal infection by *Metarhizium anisopliae* was associated with both enhanced and impaired learning in honey bees, depending on a variety of other variables, including infection with *Nosema apis*. Here, we test directly whether *Nosema* spp. parasitism affects learning and memory in honey bees.

*Nosema apis* was the historic species infecting *A. mellifera* honey bees^17^, but probably early in this century, *N. ceranae* became an invasive parasite of *A. mellifera*, transferring from Asian honey bees *A. cerana*^18^,^19^,^20^,^21^,^22^,^23^. Currently, *N. ceranae* essentially matches *N. apis*’s nearly global distribution^22^, and the two species can co-infect honey bees^9^,^22^,^24^,^25^. Some theory predicts that co-infections select for increased virulence because of within-host competition for resources^26^,^27^,^28^. Although co-infections occur, *N. ceranae* has become the predominant species in many regions^12^,^21^,^29^,^30^, which suggests that *N. ceranae* may be a better competitor than *N. apis*^24^,^31^. This raises questions about the nature of differences between the two species; there is ongoing debate about which species is more virulent^32^,^33^,^34^, which could relate to competitive ability and explain higher mortality caused by *N. ceranae* than *N. apis*^22^,^30^.

Mortality from parasites may be a direct consequence of pathology to a host, or indirect wherein behaviour is modified. For example, parasitic infections can impair cognition, both in vertebrate^35^,^36^,^37^,^38^ and invertebrate hosts^39^,^40^,^41^,^42^. A Pavlovian classical conditioning model can be used to assess honey bee learning and memory^43^,^44^,^45^. The proboscis extension reaction (usually called a reflex; PER) is a sensory physiology paradigm in which honey bees learn to associate a neutral or conditioned stimulus (e.g., odour), with an unconditioned stimulus (e.g., sucrose). Learning is assumed when the conditioned stimulus elicits an extension of the proboscis^43^,^44^,^45^,^46^; under natural conditions the proboscis must be extended to enable a honey bee to drink. Memory is tested during extinction trials in which the conditioned stimulus is presented without the unconditioned stimulus.

Effects of *N. apis* and *N. ceranae* infections, acting singly or in co-infections, on honey bee learning and memory have not been assessed previously, although one study^47^ reported on reduced homing ability in *N. ceranae*-infected bees. We used PER to test if learning and memory were compromised in honey bees infected with *Nosema*, and if there were differences among *N. apis*, *N. ceranae*, and co-infections. Based on a hypothesis of increased virulence in co-infections, we predicted that honey bee learning and memory would be most significantly affected in bees infected with both *N. apis* and *N. ceranae*. Additionally, if *N. ceranae* is more virulent than *N. apis*, we predicted the former to have a more significant effect on learning and memory. We also tested whether greater infection intensity (spores per bee, hereafter spore intensity) had greater effects on learning and memory. Tests were performed 7 and 14 days post-inoculation (d.p.i.) to evaluate whether learning and memory were affected to a greater extent later on in infections as a consequence of cumulative pathology.

## Results

### General observations

In total, 577 honey bees were conditioned using PER; some mortality occurred before bees were ready for experiments, but sample sizes were roughly equal for each treatment. PCR-testing on 109 honey bees confirmed that no cross-contamination had occurred and that all co-infections were indeed co-infections. Few honey bees (N = 3, < 1%) responded spontaneously to geraniol (i.e., PER at first exposure to odour); these were removed from statistical analyses. Thirty-three percent (190 of 577) bees did not perform PER once (non-responders) during conditioning trials. Non-responders are usually assumed to have not learned associations, and are thus not tested in extinction trials^48^,^49^,^50^ (also see discussion in ref. 44).

Mean spore counts (in millions) by treatment 7 d.p.i were 0.1 in controls, 3.2 for *N. apis*, 2.0 for *N. ceranae*, and 2.7 in coinfections. Equivalent numbers 14 d.p.i. were 0.2, 23.7, 19.1, and 22.5. Spores observed in 29 control bees 7 d.p.i. and 23 control bees 14 d.p.i. were likely experimental artefacts that regularly arise in microscope work^51^.

### Effects of treatments and spore intensities on learning and memory

**Figure 1:**
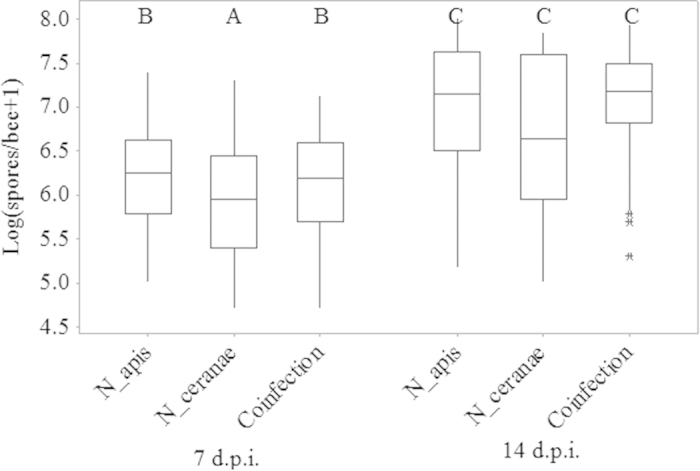
Log-transformed spore intensities in each *Nosema* treatment at 7 (left) and 14 (right) d post-inoculation. Sample sizes are given in Table 1. Treatments sharing letters were not statistically different (Mann-Whitney U tests). Boxplots show interquartile range (box), median (horizontal line within box), data range (vertical line above and below box), and outliers (asterisks).

At 7 d.p.i, spore intensities differed significantly among *Nosema* spp. treatments (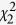 = 7.4, P = 0.03), with the *N. ceranae* treatment having significantly lower spore intensities than either *N. apis*- or co-inoculated bees (Fig. 1). Spore intensities increased from 7 d.p.i to 14 d.p.i. in all *Nosema* treatments (all Kruskal-Wallis 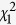 > 17.5, all Ps < 0.0001). At 14 d.p.i, there were no significant differences in spore intensities among treatments (Fig. 1).

**Figure 2:**
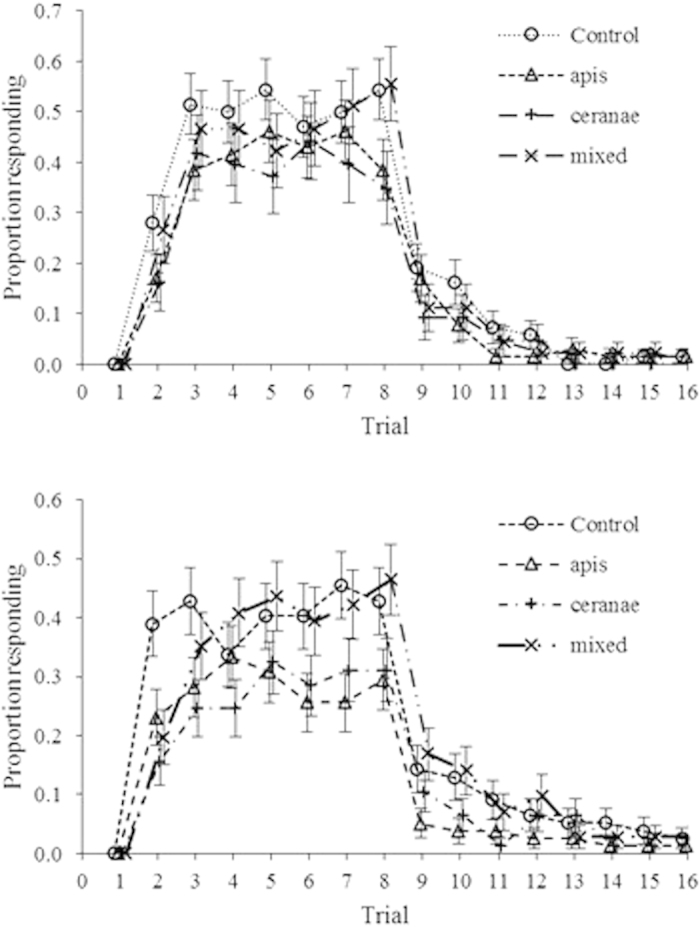
Proportion of honey bees responding to odour presented with a sucrose reward for (conditioning trials 1–8) and to odour presented without a reward (extinction trials 9–16) relative to treatment. Sample sizes are given in Table 1.

There were no significant differences in learning or memory (both indexed by the number of positive PER responses; see methods) among treatments at either 7 or 14 d.p.i. (Table 1, Fig. 2). When all honey bees were pooled, learning 14 d.p.i. was significantly better than at 7 d.p.i. (Kruskal Wallis 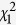 = 8.8, *P* = 0.003), but this pattern was not significant within treatment groups (all 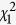 < 2.7, all *P* > 0.10). When all honey bees were pooled, there was no significant difference in memory between bees tested at 7 versus 14 d.p.i (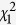 < 0.1, *P* = 0.95), nor were there differences within treatments (all 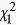 < 2.3, all *P* > 0.13).

Within *Nosema* treatments, two of 12 correlations between spore intensity and learning and memory were significant: *N. apis*-infected bees with higher spore intensities learned better 14 d.p.i. whereas co-inoculated honey bees had reduced memory 14 d.p.i. if they had higher spore intensities (Table 2).

## Discussion

Initially *N. ceranae* inoculations produced lower spore intensities compared to the other *Nosema* treatments, but spore intensities were equivalent among *Nosema* treatments by 14 d.p.i. Regardless, we found no differences in learning or memory among treatments either at 7 or 14 d.p.i., at least within the PER paradigm we used. We did observe better learning with higher spore intensities within *N. apis*-infected bees at 7 d.p.i., and poorer memory within co-inoculated bees with increased spore intensity at 14 d.p.i. The former result may indicate greater hunger and therefore more responsiveness^52^ whereas the latter result supports the hypothesis that co-infections result in increased virulence^26^. In 10 other tests, we observed no significant effects of spore intensity, so that on the whole we obtained limited evidence of effects of *Nosema* on learning and memory in honey bees, at least as assessed by PER.

Effects on learning and memory may vary with age, caste (e.g., nurse bee versus forager), satiation level, nutrition, experience, and genotype^40^,^52^,^53^. Moreover, differences in responsiveness between two genotypic strains can occur within 0 to 2 d of emergence. However, bees in this study were all of the Buckfast genetic strain, were sampled from the same colonies, emerged within a day or two of each other, received the same food, and experienced similar conditions in the cage prior to PER trials and in the trials themselves. Thus, we reduced potential influences on responses to rewards and increased our ability to detect possible effects on learning and memory.

Learning and memory were not significantly related to spore intensity among or within treatments in bees 7 d.p.i. One explanation for this is that *Nosema* spp. spores have not reached pathological levels by 7 d.p.i. This may explain why we observed no effects of spore intensity on honey bee learning and memory at 7 d.p.i., but did find some effects at 14 d.p.i.

Honey bees were tested at 7 and 14 d.p.i. to provide a range in spore intensity on which to test learning and memory and to evaluate effects of cumulative pathology. Temporal patterns we observed in spore intensities were consistent with other studies^22^,^24^. At 12 d.p.i., Forsgren and Fries (ref. 24) found *N. ceranae* and *N. apis* spores had roughly equal intensities, possibly due to lack of space for more spores in the ventriculus^54^. There is mixed evidence for whether one species of *Nosema* has a competitive advantage in co-infections^30^,^31^,^54^.

Reduced learning and memory could arise if parasites interfere with neural signalling processes^40^. Others^55^,^56^ have found that increased *Nosema* spp. spore intensity in bees was associated with energetic stress, which could affect neural signalling. In any case, increased consumption of food in response to parasitic infection is not uncommon in insects^56^ (but see ref. 57 for a review of vertebrates wherein anorexia is the dominant response to parasite infection). In previous studies, co-infected honey bees were significantly more responsive to sucrose and consumed significantly higher amounts, indicating increased appetite and overall hunger^55^,^56^.

Parasitism can disrupt ecologically significant components of cognition in animals^36^,^37^,^38^,^58^,^59^ and impairments to learning and memory could have significant detrimental effects on honey bee colony survival. However, our results provide only limited and contradictory evidence that *Nosema* spp. infections have damaging effects on learning and memory.

## Methods

### Source of spores

When spores are frozen, *N. apis* has higher rates of infectivity than *N. ceranae*^60^,^61^; therefore, frozen spores were used only to generate fresh spore stock for experimental inoculations (additional details in ref. 61). A spore homogenate was created from naturally infected dead, frozen honey bees collected in eastern Canada. Abdomens of 50 bees were added to 50 mL of distilled water and crushed using a mortar and pestle^61^. Homogenate suspensions were vortexed and viewed under phase-contrast light microscopy using a haemocytometer to count spores^62^,^63^ (Hausser Bright-Line, 1/400 cm, 0.1 mm depth). Homogenate was diluted with distilled water and assessed repeatedly using a haemocytometer until an equal spore amount of 125,000 spores per μl was achieved for each *Nosema* species. Duplex R-T PCR was performed following reference 25 to confirm species. Burgher-MacLellan *et al.*’s^25^ protocol allows one to distinguish *N. apis*, *N. ceranae*, and co-infections.

### Source of honey bees for generation of fresh spore stock

Honey bees were collected from a colony in Coldbrook, Nova Scotia, Canada. Fifty honey bees were collected from hive entrances to first confirm that a colony was free of *Nosema* spp. To verify this, honey bees were freeze-killed, suspensions of their tissues created, and *Nosema* spp. spores counted, using a haemocytometer as above. In addition to being *Nosema* spp.-free, colonies had not been treated chemically against *Nosema* spp. or Shorten to *V. destructor* mites, limiting potential for chemotherapies to affect learning or memory^64^.

After confirming that honey bees were *Nosema* spp.-free, newly emerged honey bees from the same colony were used to generate fresh, even-aged spore stock for experimental inoculations^65^. A frame with brood that was 2–3 d before eclosion was placed in a mesh bag in a nucleus box and immediately transferred to a temperature-controlled and humidity-controlled growth chamber, maintained at 33^o^ C and 45 ± 2% RH^66^.

Approximately 50 newly emerged honey bees were placed into each of two rectangular 17 × 12 × 13 cm plywood cages with removable Plexiglas sides and a wire mesh top. Honey bees were provided sucrose solution (50% w/w in water) administered *ad* libitum through a plastic syringe suspended from the wire mesh top of the cage. Food was removed after 2 d and honey bees were starved overnight in preparation for inoculations^30^.

### Inoculation of honey bees to generate fresh spore stock

Honey bees were cooled in cages for ease of handling, grasped by the thorax with tweezers, and individually fed 5 μl of 50% w/w sucrose in water solution containing 125,000 spores per μl of either *N. apis* or *N. ceranae*^61^,^67^. After force-feeding, honey bees were fed *ad libitum* on sucrose solution and, in the following days, individual honey bees were selected, freeze-killed, and spore species confirmed as above. New spore homogenate was kept at room temperature for no more than an hour and used to inoculate experimental honey bees. Fresh spores were obtained for subsequent inoculations by crushing honey bees that had gone through PER trials (see below).

### Preparing honey bees for PER testing

Frames with capped brood were collected according to previously described techniques on four occasions between July and September 2010 to provide newly emerged honey bees for inoculation and PER testing. At each occasion, after emergence, 20 honey bees were transferred to one of eight cages (same construction as above) with two cages [7- and 14-d post-inoculation (d.p.i.) honey bees] for each of four treatment groups: control (uninoculated), *N. apis*, *N. ceranae*, or co-inoculation. Honey bees were provided sucrose *ad libitum* for 2 d and then starved overnight in preparation for inoculations. Treatment groups were force fed 3 μl sucrose solution and 2 μl spore solution containing equal numbers of fresh spores as described above, achieved by dilutions^41^,^53^,^68^,^69^. The co-inoculated group received 1 μl each of *N. apis* and *N. ceranae* combined with 3 μl sucrose solution. Control honey bees were given sucrose solution to feed on *ad libitum*^53^. All cages were kept in a growth chamber as described above.

### Conditioning and extinction trials

PER trials were run both 7 and 14 d.p.i. to assess learning and memory. Food was removed from cages the night before testing and the following morning, honey bees were cooled in their cages in a −20 ^o^C freezer^70^, just until no visible signs of movement could be detected. Each honey bee was then loaded into a modified 1000-μl clear pipette tip with the tapered end removed and a small piece of wax securing the honey bee in place, so only the head was exposed and antennae and mouth parts were free to move^70^. Honey bees were randomly selected relative to parasite treatment, and PER was evaluated blind to treatment to avoid bias^71^.

Each honey bee’s PER responsiveness to sucrose was checked by applying 1.5 M sucrose solution, delivered on a wooden toothpick, to its left antenna^72^,^73^. A honey bee with a positive PER response (extension of proboscis) was fed sucrose for 3 sec and then left in darkness for 3 h. Honey bees that failed to respond were not used further in PER testing^72^,^73^,^74^.

Each bee received continuous air flow for 15 sec to acclimate it to mechanosensory stimulation. A manual valve controlled continuous air flow and delivery of the stimulus odour; both united at a mixing chamber positioned 10 to 15 mm in front of a honey bee’s head. A vacuum system behind the honey bee continuously removed odour from the testing area and contributed to drawing air and odour over honey bee antennae. Air was dispensed at a rate of approximately 1.0 L per min. Honey bees that spontaneously extended their proboscis in the first trial of the learning phase (air flow or before presentation of sucrose) were taken out of the experiment because this response indicates a previously established odour/reward association^70^,^75^. In each conditioning trial, honey bees were presented with the odour geraniol followed by a sucrose reward. Geraniol is common in many plant oils and is produced by the Nasonov gland and used as an attraction signal in worker bees^72^,^76^. Additionally, floral odours are learned faster than other odours^77^ and thus, are often used in conditioning experiments. The conditioned stimulus was prepared by pipetting 3 μl of geraniol onto filter paper that was housed in a syringe^44^,^78^,^79^,^80^.

Immediately following the 15-sec acclimation period, the conditioned stimulus was delivered for 6 sec. Three seconds after the onset of odour, sucrose (unconditioned stimulus) was delivered to the left antenna using a wooden toothpick for 1 sec and then to the proboscis for 2 sec of feeding^70^,^72^. A positive PER was recorded when the mandibles opened and the proboscis extended in response to the odour but before sucrose delivery; this indicated a learned response. The interval between two successive trials was 9 min during which time we tested the other bees that had been prepared. There were 8 conditioning trials/bee so that a score of 8 indicated maximum learning. Following 8 trials, honey bees were fed to satiation and kept in darkness at room temperature for 24 h. After these 24 h, 8 extinction trials were done to test memory; these were the same as conditioning trials but without a sucrose reward.

Individuals were scored for the number of times they exhibited PER in response to the odour in conditioning and extinction trials. Following extinction trials, honey bees were freeze-killed and spore counts carried out as above. Conventional PCR was completed on all honey bees in the co-inoculated treatment and a random sample of *N. apis* and *N. ceranae* treatments to confirm that no cross-contamination had occurred.

### Statistical analyses

Statistical analyses were conducted in SAS version 9.3 (Cary, North Carolina). Data were not normally distributed (Kolmogorov-Smirnov tests for normality) even after transformations; thus, non-parametric tests of raw data were done. Spore intensities, learning, and memory were compared among treatments using Kruskal-Wallis tests, following up with Mann-Whitney U tests where significance was obtained. We tested whether learning and memory were related to spore intensity using Spearman’s rank correlations. Spore intensities, learning, and memory were also compared between 7 and 14 d.p.i. using Kruskal-Wallis tests.

## Additional Information

**How to cite this article**: Charbonneau, L. R. *et al.* Effects of *Nosema apis, N. ceranae*, and coinfections on honey bee (*Apis mellifera*) learning and memory. *Sci. Rep.* **6**, 22626; doi: 10.1038/srep22626 (2016).

## Figures and Tables

**Table 1:** Learning and memory (mean number of positive PERs in 8 trials for all bees within a treatment) did not differ among treatments 7 or 14 d post-inoculateion.

Variable	Days post-inoculation	Treatment								Kruskal-Wallis	
		Control		*N. apis*		*N. ceranae*		Co-inoculation		statistics	
		*N*	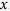	*N*	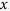	*N*	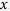	*N*	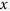	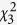	*P*
Learning	7	89	2.7	85	2.2	80	2.2	83	2.6	2.9	0.40
Learning	14	68	3.4	64	2.7	38	2.6	46	3.2	4.7	0.20
Memory	7	82	0.6	62	0.5	65	0.6	69	0.8	2.9	0.41
Memory	14	58	0.6	48	0.5	28	0.3	37	0.4	5.1	0.17

*N* is total number of bees tested in each treatment (for 8 conditioning and 8 extinction trials).

**Table 2:** Within treatment Spearman correlations between spore intensities and learning and memory.

Variable	Days post-inoculation	*Nosema apis*			*Nosema ceranae*			Co-inoculation		
		N	r_s_	p	N	r_s_	p	N	r_s_	p
Learning	7	85	−0.08	0.47	80	0.10	0.07	83	0.09	0.43
Memory	7	82	0.02	0.86	65	0.39	0.59	69	<0.01	0.99
Learning	14	64	**0.21**	**0.02**	38	0.14	0.39	46	0.09	0.57
Memory	14	48	0.10	0.90	28	0.11	0.56	37	**−0.32**	**0.05**

Significant results in bold.
